# A meta-analysis on first-trimester blood count parameters—is the neutrophil-to-lymphocyte ratio a potentially novel method for first-trimester preeclampsia screening?

**DOI:** 10.3389/fmed.2024.1336764

**Published:** 2024-04-03

**Authors:** Balázs Mészáros, Dániel S. Veres, Luca Nagyistók, Bence G. Kovács, Zoltán Kukor, Sándor Valent

**Affiliations:** ^1^Department of Obstetrics and Gynecology, Semmelweis University, Budapest, Hungary; ^2^Department of Biophysics and Radiation Biology, Semmelweis University, Budapest, Hungary; ^3^Dél-Pest Centrum Hospital National Hematology and Infectious Diseases Institute, Budapest, Hungary; ^4^Department of Molecular Biology, Institute of Biochemistry and Molecular Biology, Semmelweis University, Budapest, Hungary

**Keywords:** NLR, neutrophil-to-lymphocyte ratio, first trimester, pre-eclampsia, preeclampsia screening

## Abstract

**Objective:**

Meta-analysis focusing on the role of first-trimester neutrophil-to-lymphocyte ratio (NLR) in the prediction of preeclampsia.

**Data sources:**

PubMed, Scopus, Web of Science, Cochrane Library, and Embase databases were queried from inception up to December 31, 2022.

**Study eligibility criteria:**

The study included all types of original research that was conducted in humans and values of NLR were measured during the first trimester, among patients who later developed preeclampsia, compared to the values of control groups.

**Study appraisal and synthesis methods:**

Two reviewers independently performed data abstraction and quality appraisal, and disagreements were resolved by consensus and, if necessary, by the opinion of a third reviewer. During the analysis, PRISMA and MOOSE guidelines were followed. All statistical analyses were made with *R*.

**Results:**

For the research on the predictive role of NLR values in the first trimester for preeclampsia, a total of 6 studies were selected for analysis, covering 2,469 patients. The meta-analysis revealed a 95% confidence interval (CI) for the effect size of 0.641 to 1.523, with a prediction interval of 0.027 to 2.137.

**Conclusion:**

Based on the analysis, NLR is a promising biochemical marker for future pieces of research that try to find new screening methods for first-trimester preeclampsia. We encourage other researchers to examine NLR’s predictive value combined with other markers in preeclampsia screening, this way being able to find new and affordable protocols for first-trimester preeclampsia screening.

**Systematic review registration:**

identifier CRD42023392663.

## 1 Introduction

Preeclampsia is a pregnancy-specific disorder, and it was defined for decades by the new onset of hypertension and proteinuria. According to the latest guidelines such as NICE (National Institute for Health and Care Excellence) and ISSHP (International Society for the Study of Hypertension in Pregnancy) proteinuria is not mandatory for the diagnosis of preeclampsia: according to NICE -preeclampsia is characterized by the onset of new hypertension after 20 weeks of pregnancy, accompanied by one or more newly emerging features: these features may include substantial proteinuria or maternal organ dysfunction, such as renal insufficiency, liver involvement, neurological complications, or hematological complications (1, 2). By the definition of - ISSHP, which closely resembles NICE’s definition—pre-eclampsia is diagnosed when new-onset hypertension (systolic blood pressure >140 mmHg, diastolic blood pressure >90 mmHg) occurs after 20 weeks of pregnancy, accompanied by at least one additional symptom or group of symptoms, which may include: proteinuria; dysfunction of other maternal organs (such as liver, kidney, central nervous system); hematological abnormalities; uteroplacental dysfunction (e.g., intrauterine growth restriction—IUGR, and/or abnormal Doppler ultrasound results concerning uteroplacental circulation) (3). Preeclampsia affects 2–8% of pregnant women and is one of the leading causes of maternal and neonatal morbidity and mortality in the world, particularly in low-income countries (4–6). According to WHO, in developing countries, 16% of maternal deaths are attributed to hypertensive disorders, and the reduction of maternal mortality is a global goal (7, 8). Despite its significant impact on obstetrics and healthcare in general, preeclampsia has remained an enigmatic field of medicine. However, recently, new preventive and screening methods have been tested (9).

The early identification of patients at high risk for preeclampsia can be crucial for achieving significantly improved maternal and perinatal outcomes. This involves providing closer surveillance, considering prophylactic use of low-dose aspirin therapy, administering antihypertensive medications, and opting for earlier induced delivery (10, 11).

Since inflammatory reactions are suggested behind the pathomechanism of preeclampsia (12–16) in recent years publications have been evaluating the role of white blood cells both in clinical studies and animal models in the prediction of preeclampsia (17, 18). The distribution of white blood cells can be monitored through the neutrophil-to-lymphocyte ratio (NLR), which has been found to be a useful marker for inflammatory diseases such as systemic lupus erythematosus (SLE), spondyloarthritis, psoriasis, psoriatic arthritis, various types of tumors, and Takayasu arteritis (TA) (19–25). There have also been studies that evaluated the role of NLR in pregnancy-related diseases (26, 27). Moreover, in recent years, several meta-analyses have been published that found elevated NLRs in blood samples from mothers who experienced preeclampsia (28, 29).

The fact that laboratory findings are widely affordable and accessible even in developing countries (30, 31) and neutrophil and lymphocyte counts are usually part of routine laboratory tests (32) are other reasons why NLR would provide beneficial predictive value in preeclampsia.

## 2 Object

This current meta-analysis aims to evaluate the role that first-trimester NLR values can play in preeclampsia screening.

## 3 Methods

### 3.1 Eligibility criteria, information sources, search strategy

The data for the meta-analysis were collected by two independent researchers from PubMed, Scopus, Web of Science, Cochrane Library, and Embase databases. Disagreements were resolved through consensus and, if necessary, by the opinion of a third reviewer. The database searches were conducted until December 31, 2022, without any additional time restrictions. Language restrictions were not applied.

For the preparation and planning of this analysis, a PRISMA checklist and the MOOSE method were utilized (33, 34).

### 3.2 Study selection

For this research, the keywords “NLR” supplemented with “preeclampsia” were used. Each search was conducted across five online medical databases: PubMed, Cochrane Library, Scopus, Embase, and Web of Science. During the screening process, the research group aimed to select studies that reported NLR values in the first trimester of pregnancy in women who later developed preeclampsia. These values were compared to control groups consisting of women who remained normotensive and free of obstetrical complications during their pregnancies.

### 3.3 Data extraction

From the studies collected for further review, the following data were extracted: the study objective; the number of mild preeclamptic patients included in the study; the number of severe preeclamptic patients included in the study; the total number of preeclamptic patients included in the study; the number of control (healthy, normotensive) pregnant patients; the time of data collection (trimester, weeks); NLR values of mild preeclamptic patients and their corresponding standard deviations; NLR values of severe preeclamptic patients and their corresponding standard deviations; NLR values of preeclamptic patients and their corresponding standard deviations; NLR values of healthy, normotensive patients (control group) and their corresponding standard deviations; and *p*-values. Additionally, both researchers collected the articles’ titles, authors, publication years, publishers, and DOIs.

### 3.4 Assessment of risk of bias

The Newcastle–Ottawa scale (NOS) (35) was used to assess the quality of the included studies. The quality assessment was conducted independently by two authors, with any disagreements resolved through consensus or, if necessary, by involving a third author. The NOS evaluates articles based on three main factors: the selection of study groups, the comparability of groups, and the ascertainment of exposure, assigning scores ranging from 0 to 9. A score of 0 represents the worst possible quality, while 9 indicates the best possible quality. Studies scoring 0–4 stars are considered low quality, while those receiving 5 or more stars are deemed moderate to high quality. According to the authors, all the included articles received 6 or more stars on the NOS.

### 3.5 Data synthesis

Mean difference (MD) with a 95% confidence interval (CI) was used to express the effect size. To calculate the mean difference the number of patients, the mean, and standard deviation (SD) of the variable of interest for the “preeclampsia” and “without preeclampsia” (i.e., control) groups were extracted from the studies. The mean difference is calculated as the mean of the “preeclampsia” group minus the mean of the “without preeclampsia” group. In some cases (highlighted with * in the forest plots) means and SDs were given for moderate and severe preeclampsia subgroups separately and we combined them using established formulae https://training.cochrane.org/handbook/archive/v6.1/chapter-06#section-6-5-2-1 (36).

As we anticipated considerable between-study heterogeneity, a random-effects model was used to pool effect sizes. The inverse variance weighting method was used to calculate the pooled mean difference. Hartung-Knapp adjustment (37, 38) was applied as the study number and sample sizes were relatively small. To estimate the heterogeneity variance measure (tau squared), a restricted maximum-likelihood estimator was applied with the Q profile method (39). Additionally, between-study heterogeneity was described by Higgins and Thompson’s (*I* squared) statistics (40). Forest plots were used to graphically summarize the results. The confidence interval of each individual study was calculated based on the *t*-distribution. Additionally, where applicable, we reported prediction intervals (i.e., the expected range of effects of future studies) of results following the recommendations of IntHout et al. (41).

Outlier and influence analyses were carried out following the recommendations of Harrer et al. (42) and Viechtbauer and Cheung (43). Publication bias was assessed with Egger’s test (at a significance level of 10%) (44)—however, results should handled critically due to the small number of studies.

All statistical analyses were made with *R* software (45) using the meta package (46) for main calculations, and the dmetar package (47) for influential analysis.

## 4 Results

### 4.1 Study selection

#### 4.1.1 Study selection for evaluating NLR’s predictive role in preeclampsia

**Figure 1:**
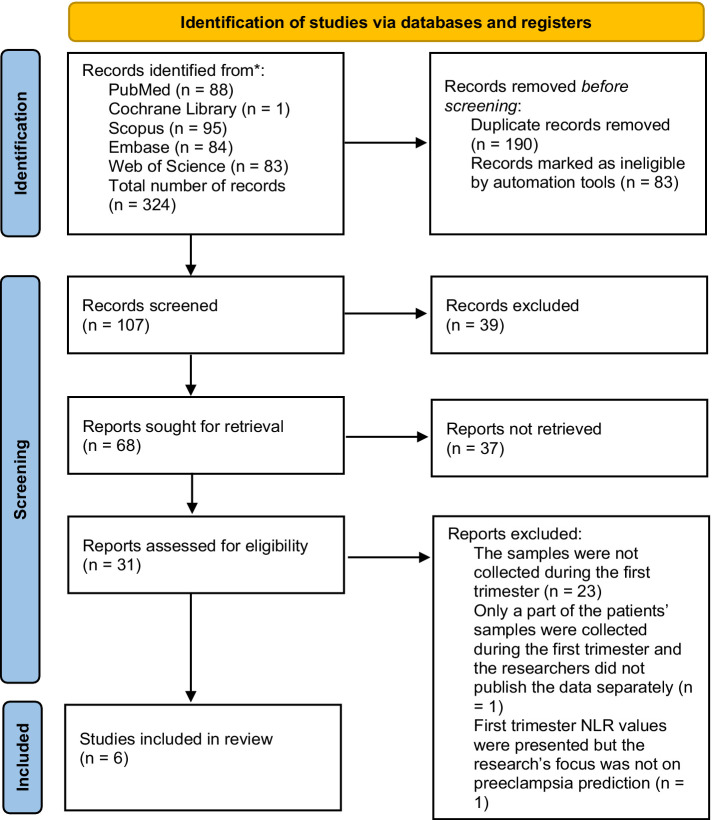
Selection of the studies for the analysis of NLR values’ predictive role in first-trimester preeclampsia.

For the research, the keywords “NLR” and “preeclampsia” were combined, and searches were conducted in 5 online medical databases (PubMed, Cochrane Library, Scopus, Embase, Web of Science). In total, 324 articles were found, and 134 remained after removing duplicates. An additional 103 articles were excluded because they were irrelevant to the conducted research. In our meta-analysis, we aimed to find clinical studies that utilized first-trimester NLR values as predictive markers of preeclampsia. We excluded studies that were not clinical (e.g., systematic reviews, meta-analyses), letters to other publications, and clinical studies that focused on NLRP3 (NOD-, LRR-, and pyrin domain-containing protein) 3 values in pre-eclamptic women, studies which were results for our searches because they used negative likelihood ratio which’s short form is also NLR and the studies which were clinical but did not use first trimester NLR findings. The remaining 31 pieces of research were selected for detailed screening and out of these 25 got excluded because the samples were not collected during the first trimester (23 studies), only a part of the patients’ samples were collected during the first trimester and the researchers did not publish the data separately (1 study) or first trimester NLR values were presented but the research’s focus was not on preeclampsia prediction (1). The PRISMA flow diagram was conducted regarding strictly The PRISMA 2020 statement: an updated guideline for reporting systematic reviews (34) (Figure 1). Of the remaining 6 (48–53) pieces of research the data (the NLR values in the control and preeclampsia groups and their standard deviations) got extracted for the meta-analysis.

### 4.2 Study characteristics

In our study, we included overall 6 studies, the number of preeclampsia patients, the number of the control groups, the ages of the patients (both mean and standard deviation) and the BMIs of the patients (both mean and standard deviation), gestational age at delivery (both mean and standard deviation), are presented on Table 1.

**Table 1:** Studies included in the meta-analysis.

	Study ID	Sample size		Age		BMI		Gestational age at delivery	
		Preeclampsia	Control	Preeclampsia	Control	Preeclampsia	Control	Preeclampsia	Control
1	Gezer et al. (51)	209	221	26.6 ± 6	25.8 ± 4.9	25.7 ± 3.7	25.2 ± 4.1	35.8 ± 3.02	39.37 ± 1.16
2	Hale et al. (53)	214	240	28.7 ± 3.4	27.5 ± 3.5	22.9 ± 3.2	22.7 ± 3.5	37.6 ± 1.1	40.5 ± 1.5
3	Kirbas et al. (50)	614	320	Severe PE: 29.3 ± 14.3, mild PE: 27.9 ± 4.9	27.0 ± 5.0	Severe PE: 23.7 ± 3.6, mild PE: 22.9 ± 3.1	22.7 ± 3.6	Severe PE: 33.0 ± 3.5, mild PE: 37.5 ± 2.1	40.6 ± 1.6
4	Oğlak et al. (49)	201	100	Severe PE: 28.7 ± 6.8, mild PE: 28.3 ± 7.4	27.4 ± 6.1	NR	NR	NR	NR
5	Bulbul et al. (48)	161	161	30.91 ± 6.47	30.08 ± 6.04	28.00 ± 2.62	26.73 ± 2.97	36.4 ± 2.9	38.2 ± 1.9
6	Mannaerts et al. (54)	14	14	29 (no SD presented)	31 (no SD presented)	26.7 ± 3.4	28.0 ± 3.6	NR	NR

### 4.3 Risk of bias

As mentioned above, publication bias was assessed with Egger’s test (at a significance level of 10% due to the small study number).

#### 4.3.1 Bias in NLR research

Although Egger’s test *p*-value is 0.2132, the meta-analysis contains few studies therefore Egger’s test may lack the statistical power to detect bias or it could give a false “positive” result.

### 4.4 Synthesis of results

#### 4.4.1 NLR results

A total of 6 studies were selected for analyses covering a total of 2,469 patients.

On average, the effect size is 1.082. The 95% confidence interval of the effect size is 0.641 to 1.523, which tells us that the mean effect size in the universe of comparable studies could fall in this range.

The between-study heterogeneity expressed as *I*^2^ value is 0.765 (95% CI, 0.473–0.895), which tells us that 76.5% of the variance in observed effects reflects variance in true effects rather than sampling error. The variance of true effects *Τ*^2^ is 0.12 and the standard deviation of true effects *Τ* is 0.34.

**Figure 2:**
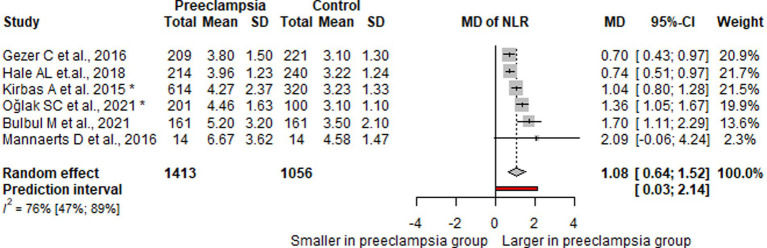
Forest plot of NLR values.

The prediction interval is 0.027 to 2.137. Based on that we would expect in some 95% of all populations comparable to those in the analysis, the true effect size will fall in this range (Figure 2).

## 5 Comment

### 5.1 Principal findings

NLR’s prediction interval fell in the range of 0.027 to 2.137, and the 95% confidence interval of the effect size is 0.641 to 1.523, all the evaluated studies found elevated levels of NLR in mothers who later during their pregnancies developed preeclampsia.

### 5.2 Comparison with existing literature

#### 5.2.1 The possible explanation behind the elevation of NLR in preeclampsia

Recent studies show that IL-6, IL-8, and IL-17 play an important role in preeclampsia and the production of neutrophil (55–58). One of IL-8’s most important roles is the attraction of neutrophils to the inflamed areas, they play a role in neutrophil recruitment to the endometrium (this way contributing to preeclampsia development), and IL-8 also stimulates neutrophil degranulation (59–62). While IL-6 is linked to genes that stimulate the proliferation, maturation, and activation of neutrophils (63–67). Levels of IL-17A are elevated in preeclampsia and it stimulates the expression of neutrophil chemokines in vascular smooth muscle, IL-17A also increases the levels of G-CSF and GM-CSF which both increase the production of neutrophils (57, 68, 69).

#### 5.2.2 The neutrophil-to-lymphocyte ratio in clinical research

NLR is more and more getting at the center of new studies: while in the PubMed database for the search “neutrophil-to-lymphocyte ratio” there are 65 results from 2012, this number was 1,669 in 2022.

NLR is also studied as a predictive biomarker in COVID-19 (70–72): Fernandes et al. (73) found that NLR levels are higher in COVID-19 patients who needed invasive mechanical ventilation than the control group of COVID-19 patients who did not require invasive mechanical ventilation.

NLR was studied in pregnant COVID-19 patients as well (74, 75): Aydin Güzey et al. (76) evaluated 254 cesarean sections with COVID-19 and found elevated levels of NLR among the symptomatic patients. Our research group also presented a case report where we found elevated NLR in a preeclamptic COVID-19 patient (77). Our research group additionally conducted a case–control study, which included 45 pregnant patients with COVID-19. Statistical analyses revealed that NLR values were notably elevated in patients who succumbed to fatal COVID-19 compared to those who survived the disease (78).

Even though Lurie et al. (79) published their results as early as 1998 of growing neutrophil counts and declining lymphocyte counts in preeclamptic patients, they did not try to evaluate the quotient of these data in PE screening. The first study on NLR’s predictive role in preeclampsia was published in 2015 by Kurtoglu et al. (80) and since then a handful of other studies were published evaluating NLR’s role in all the 3 trimesters (54, 81, 82).

It is important to mention Kang’s et al. (29) meta-analysis from 2019, which found that NLR levels are higher in symptomatic preeclamptic patients compared to control groups. Their meta-analysis also suggests that NLR values can be used to evaluate disease severity. Despite the existence of this prior meta-analysis, our work provides valuable insights as we aimed to evaluate NLR values in the first trimester, before the onset of preeclampsia. Therefore, our study assesses the potential role of NLR values in the screening of preeclampsia. Furthermore, our meta-analysis was justified because, nearly 4 years after their study, numerous new clinical studies have emerged investigating NLR in relation to preeclampsia. However, to the best of our knowledge, there is still no meta-analysis that specifically evaluates the role of this laboratory marker purely in preeclampsia prediction.

#### 5.2.3 Preeclampsia’s first-trimester detection, its importance

As preeclampsia is a relatively common clinical syndrome of the human pregnancy, with a prevalence of 2–8% (5), its only definitive treatment currently the termination of the pregnancy: the delivery of the placenta and the neonate (83) and remains one of the leading causes of maternal- and neonatal morbidity (84) it is eager to find more and more accurate screening methods and therapies.

Large cohort studies and meta-analyses indicate that the main risk factors for preeclampsia development are obesity, antiphospholipid antibody syndrome, chronic hypertension, pregestational diabetes, the use of assisted reproductive technology, nulliparity, and irregular antenatal visits (85–87).

In the screening of preeclampsia, the evaluation of maternal characteristics (maternal age, weight, height, ethnicity, and smoking), medical (chronic hypertension, diabetes, family history of preeclampsia), and obstetrical history (prior pregnancies affected by preeclampsia) is key in the risk calculation of preeclampsia (88, 89). The two most frequently used guidelines that aim to stratify risk using maternal risk factors and characteristics are the American College of Obstetricians and Gynecologists (ACOG) and the National Institute for Health and Care Excellence (NICE) (1, 90). However, the use of risk factors for first-trimester preeclampsia screening performs with poor sensitivity (91).

Another important basis of preeclampsia screening is the usage of Doppler ultrasound, in which case MAP (mean arterial pressure) and uterine artery pulsatility index (UtA-PI) are measured (88, 92).

Biochemical markers are also widely used in preeclampsia’s first-trimester screening: abnormal serum levels of placental growth factor (PlGF), pregnancy-associated plasma protein A (PAPP-A), placental growth factor (PlGF), alpha-fetoprotein (AFP), human chorionic gonadotropin (hCG), unconjugated estriol (uE3), Inhibin A, soluble-endoglin (sEng), and soluble Flt-1 (sFlt-1) are all associated with higher risks of preeclampsia (93–96).

While these methods and the combination of them keep improving it is still urgent to find new markers (possibly ones that can be applied in developing countries as well) to supplement and to make better the currently existing protocols which are key to the reduction of maternal mortality (7, 97).

As a result of our meta-analysis, we found, that to the list of useful biochemical markers, higher levels of NLR can be added: according to the studies used in our analysis, this marker is elevated in first-trimester preeclampsia, moreover, NLR is also easily and widely accessible. However, we maintain that further research should evaluate the usage of the above-mentioned biochemical and biophysical markers combined with NLR, to find more and more beneficial and affordable screening methods.

#### 5.2.4 Preventive medication for preeclampsia

We evaluated NLR’s first-trimester predictive role because we maintain that novel and more accurate screening methods could help obstetricians to detect preeclampsia earlier and consequently, start the treatment or the preventive treatment earlier.

In preeclampsia prevention, the most widely used medication is low-dose aspirin therapy (98, 99). However, there is a growing skepticism against aspirin use in preeclampsia prevention: Lin et al. found that 100 mg of aspirin per day, initiated from 12 to 20 gestational weeks until 34 weeks of gestation, did not reduce the incidence of preeclampsia in pregnant women with high-risk factors (100).

Consequently, new studies are experimenting with other treatments in preeclampsia prevention: Cruz-Lemini et al. (101) published a meta-analysis on low-molecular-weight heparin therapy in women at high risk of preeclampsia. They found that LMWH therapy significantly reduces the risk of preeclampsia and other placenta-mediated complications if the treatment is started before the 16th gestational week.

Our research group’s earlier meta-analysis highlighted that pravastatin therapy started before the 20th gestational week reduces preeclampsia development. The therapy is also beneficial for neonates, as it reduces the number of neonates born with IUGR, neonatal admissions to intensive care units, and the occurrence of preterm deliveries (102).

Calcium, magnesium, and vitamin D supplementation may also be useful in preeclampsia prevention (103–107).

As there are more and more medications that are proven to be effective in preeclampsia prevention it would be key to find more screening methods that would help doctors to detect the risk of preeclampsia earlier and define which patients would need to take preventive medications: this is another reason why we think that first-trimester NLR values in preeclampsia screening should be furtherly evaluated.

#### 5.2.5 The importance of finding cost-effective screening methods

Preeclampsia, even in the 21st century in developed countries remains one of the leading causes of maternal mortality (108–111) and it also puts a large financial burden on health care systems: in 2012, the cost of preeclampsia within the first 12 months of delivery was $2.18 billion in the United States ($1.15 billion for infants and $1.03 billion for mothers) (112). While preeclampsia is a huge and unsolved problem even in developed countries, developing countries are affected more severely (7, 113–117).

In developing countries, it is key to find cost-effective ways the screening and treatment of diseases, but the price is an important aspect in developed countries also.

As NLR is proven to be a cost-effective, relatively accessible biomarker of several diseases (118–121), and the results of our analysis also highlight it as a promising addition to first-trimester preeclampsia screening methods, we maintain that elevated levels of NLR in preeclampsia screening should be evaluated in further clinical studies.

### 5.3 Strengths and limitations

We are pleased to present our work as the first meta-analysis or systematic review examining the role of NLR in predicting preeclampsia during the first trimester of pregnancy. While previous meta-analyses (28, 29) have explored NLR in preeclampsia prediction, our study uniquely focuses on evaluating first-trimester laboratory findings. We believe that our analysis offers valuable insights into the potential utility of these values for screening preeclampsia during the first trimester. Consequently, our study contributes to a more structured understanding of this area and may serve as a foundation for future clinical investigations, both prospective and retrospective, into the use of NLR in first-trimester preeclampsia screenings.

However, we acknowledge that despite our study’s significant contribution to the field, its primary limitation lies in the small number of eligible studies and patients included. Owing to this limited pool, we faced challenges in estimating the prediction interval of true study effect sizes with a high degree of certainty. Additionally, the limited number of studies prevented us from thoroughly evaluating publication bias or conducting outlier and influential analyses.

## 6 Conclusions and implications

As the presented statistics show the effect size (1.082), the 95% confidence interval of the effect size (0.641 to 1.523), the standard deviation of true effects (0.34), and the prediction interval (0.027 to 2.137) all fall in a range that lets us conclude that NLR can have a role in first-trimester preeclampsia screening.

We encourage other researchers to examine NLR in cohort studies and randomized clinical studies, alone and combined with other screening methods to find new screening protocols for preeclampsia early on, in the first trimester during pregnancy, this way allowing prophylactic preeclampsia treatment to start earlier.

We maintain that because of the circumstances mentioned in the part “Discussions” it is desired to experiment with screening methods that are: (a) can help to detect preeclampsia early during pregnancy (b) are applicable in low-resource settings-based on our analysis NLR fulfills both criteria.

## Data availability statement

The original contributions presented in the study are included in the article/supplementary material, further inquiries can be directed to the corresponding author.

## Author contributions

BM: Conceptualization, Investigation, Writing – original draft. DV: Formal analysis, Investigation, Software, Validation, Visualization, Writing – original draft. LN: Formal analysis, Investigation, Writing – original draft. BK: Supervision, Writing – review & editing. ZK: Conceptualization, Investigation, Methodology, Supervision, Writing – review & editing. SV: Conceptualization, Investigation, Methodology, Supervision, Writing – review & editing.

## Glossary

NLR: Neutrophil-to-lymphocyte ratio
ACOG: American College of Obstetricians and Gynecologists
NICE: National Institute for Health and Care Excellence
SLE: Systemic lupus erythematosus
TA: Takayasu arteritis
DOI: Digital object identifier
NLRP3: NOD-, LRR- and pyrin domain-containing protein 3
MAP: Mean arterial pressure
UtA-PI: Uterine artery pulsatility index
PAPP-A: Pregnancy-associated plasma protein A
PlGF: Placental growth factor
AFP: Alpha-fetoprotein
hCG: Human chorionic gonadotropin
uE3: Unconjugated estriol
sEng: Soluble-endoglin
sFlt-1: Soluble Flt-1
LMWH: Low-molecular-weight heparin
IL: Interleukin
G-CSF: Granulocyte colony-stimulating factor
GM-CSF: Granulocyte-macrophage colony-stimulating factor

## References

[1] Webster K Fishburn S Maresh M Findlay SC Chappell LC Guideline Committee. Diagnosis and management of hypertension in pregnancy: summary of updated NICE guidance. BMJ. (2019) 366:l5119. doi: 10.1136/bmj.l5119, PMID: 31501137

[2] Phumsiripaiboon P Suksai M Suntharasaj T Geater A. Screening for pre-eclampsia: performance of National Institute for Health and Care Excellence guidelines versus American College of Obstetricians and Gynecologists recommendations. J Obstet Gynaecol Res. (2020) 46:2323–31. doi: 10.1111/jog.14425, PMID: 32815191

[3] Burton GJ Redman CW Roberts JM Moffett A. Pre-eclampsia: pathophysiology and clinical implications. BMJ. (2019) 366:l2381. doi: 10.1136/bmj.l2381, PMID: 31307997

[4] Ananth CV Keyes KM Wapner RJ. Pre-eclampsia rates in the United States, 1980–2010: age-period-cohort analysis. BMJ. (2013) 347:f6564. doi: 10.1136/bmj.f6564, PMID: 24201165 PMC3898425

[5] Steegers EA von Dadelszen P Duvekot JJ Pijnenborg R. Pre-eclampsia. Lancet. (2010) 376:631–44. doi: 10.1016/S0140-6736(10)60279-6 20598363

[6] Chappell LC Cluver CA Kingdom J Tong S. Pre-eclampsia. Lancet. (2021) 398:341–54. doi: 10.1016/S0140-6736(20)32335-7 34051884

[7] Khan KS Wojdyla D Say L Gülmezoglu AM Van Look PF. WHO analysis of causes of maternal death: a systematic review. Lancet. (2006) 367:1066–74. doi: 10.1016/S0140-6736(06)68397-9 16581405

[8] Nyfløt L Sitras V. Strategies to reduce global maternal mortality. Acta Obstet Gynecol Scand. (2018) 97:639–40. doi: 10.1111/aogs.13356 29726626

[9] Mészáros B Kukor Z Valent S. Recent advances in the prevention and screening of preeclampsia. J Clin Med. (2023) 12:6020. doi: 10.3390/jcm12186020, PMID: 37762960 PMC10532380

[10] Coviello EM Iqbal SN Grantz KL Huang CC Landy HJ Reddy UM. Early preterm preeclampsia outcomes by intended mode of delivery. Am J Obstet Gynecol. (2019) 220:100.e1–9. doi: 10.1016/j.ajog.2018.09.027, PMID: 30273585 PMC7605098

[11] Rolnik DL Wright D Poon LC O’Gorman N Syngelaki A de Paco Matallana C . Aspirin versus placebo in pregnancies at high risk for preterm preeclampsia. N Engl J Med. (2017) 377:613–22. doi: 10.1056/NEJMoa1704559, PMID: 28657417

[12] Michalczyk M Celewicz A Celewicz M Woźniakowska-Gondek P Rzepka R. The role of inflammation in the pathogenesis of preeclampsia. Mediat Inflamm. (2020) 2020:3864941–9. doi: 10.1155/2020/3864941, PMID: 33082708 PMC7556088

[13] Perucci LO Corrêa MD Dusse LM Gomes KB Sousa LP. Resolution of inflammation pathways in preeclampsia-a narrative review. Immunol Res. (2017) 65:774–89. doi: 10.1007/s12026-017-8921-3, PMID: 28391374

[14] Bu C Wang Z Ren Y Chen D Jiang SW. Syncytin-1 nonfusogenic activities modulate inflammation and contribute to preeclampsia pathogenesis. Cell Mol Life Sci. (2022) 79:290. doi: 10.1007/s00018-022-04294-2 35536515 PMC11073204

[15] Shamshirsaz AA Paidas M Krikun G. Preeclampsia, hypoxia, thrombosis, and inflammation. J Pregnancy. (2012) 2012:374047. doi: 10.1155/2012/374047, PMID: 22175023 PMC3235807

[16] Alston MC Redman LM Sones JL. An overview of obesity, cholesterol, and systemic inflammation in preeclampsia. Nutrients. (2022) 14:2087. doi: 10.3390/nu14102087, PMID: 35631228 PMC9143481

[17] Banerjee S Huang Z Wang Z Nakashima A Saito S Sharma S . Etiological value of sterile inflammation in preeclampsia: is it a non-infectious pregnancy complication? Front Cell Infect Microbiol. (2021) 11:694298. doi: 10.3389/fcimb.2021.694298, PMID: 34485175 PMC8415471

[18] Zeng H Han X Zhu Z Yu S Mei S Cheng X . Increased uterine NLRP3 inflammasome and leucocyte infiltration in a rat model of preeclampsia. Am J Reprod Immunol. (2021) 86:e13493. doi: 10.1111/aji.13493, PMID: 34375018

[19] Qin B Ma N Tang Q Wei T Yang M Fu H . Neutrophil to lymphocyte ratio (NLR) and platelet to lymphocyte ratio (PLR) were useful markers in assessment of inflammatory response and disease activity in SLE patients. Mod Rheumatol. (2016) 26:372–6. doi: 10.3109/14397595.2015.1091136, PMID: 26403379

[20] Gasparyan AY Ayvazyan L Mukanova U Yessirkepov M Kitas GD. The platelet-to-lymphocyte ratio as an inflammatory marker in rheumatic diseases. Ann Lab Med. (2019) 39:345–57. doi: 10.3343/alm.2019.39.4.345, PMID: 30809980 PMC6400713

[21] Wang L Wang C Jia X Yang M Yu J. Relationship between neutrophil-to-lymphocyte ratio and systemic lupus erythematosus: a meta-analysis. Clinics. (2020) 75:e1450. doi: 10.6061/clinics/2020/e1450, PMID: 32321113 PMC7153360

[22] Seringec Akkececi N Yildirim Cetin G Gogebakan H Acipayam C. The C-reactive protein/albumin ratio and complete blood count parameters as indicators of disease activity in patients with Takayasu arteritis. Med Sci Monit. (2019) 25:1401–9. doi: 10.12659/MSM.912495, PMID: 30792377 PMC6396438

[23] Seng JJB Kwan YH Low LL Thumboo J Fong WSW. Role of neutrophil to lymphocyte ratio (NLR), platelet to lymphocyte ratio (PLR) and mean platelet volume (MPV) in assessing disease control in Asian patients with axial *Spondyloarthritis*. Biomarkers. (2018) 23:335–8. doi: 10.1080/1354750X.2018.1425916, PMID: 29307233

[24] Ren Z Yang J Liang J Xu Y Lu G Han Y . Monitoring of postoperative neutrophil-to-lymphocyte ratio, D-dimer, and CA153 in: diagnostic value for recurrent and metastatic breast cancer. Front Surg. (2023) 9:927491. doi: 10.3389/fsurg.2022.927491, PMID: 36684341 PMC9853451

[25] Justesen MM Jakobsen KK Bendtsen SK Garset-Zamani M Mordhorst C Carlander ALF . Pretreatment neutrophil-to-lymphocyte ratio as a prognostic marker for the outcome of HPV-positive and HPV-negative oropharyngeal squamous cell carcinoma. Viruses. (2023) 15:198. doi: 10.3390/v15010198, PMID: 36680237 PMC9863220

[26] Sisti G Faraci A Silva J Upadhyay R. Neutrophil-to-lymphocyte ratio, platelet-to-lymphocyte ratio, and routine complete blood count components in HELLP syndrome: a matched case control study. Medicina. (2019) 55:123. doi: 10.3390/medicina55050123 31072037 PMC6572204

[27] Christoforaki V Zafeiriou Z Daskalakis G Katasos T Siristatidis C. First trimester neutrophil to lymphocyte ratio (NLR) and pregnancy outcome. J Obstet Gynaecol. (2020) 40:59–64. doi: 10.1080/01443615.2019.1606171, PMID: 31609136

[28] Zheng WF Zhan J Chen A Ma H Yang H Maharjan R. Diagnostic value of neutrophil-lymphocyte ratio in preeclampsia: a PRISMA-compliant systematic review and meta-analysis. Medicine. (2019) 98:e18496. doi: 10.1097/MD.0000000000018496, PMID: 31861035 PMC6940150

[29] Kang Q Li W Yu N Fan L Zhang Y Sha M . Predictive role of neutrophil-to-lymphocyte ratio in preeclampsia: a meta-analysis including 3982 patients. Pregnancy Hypertens. (2020) 20:111–8. doi: 10.1016/j.preghy.2020.03.009, PMID: 32279029

[30] Tsai ER Tintu AN Demirtas D Boucherie RJ de Jonge R de Rijke YB. A critical review of laboratory performance indicators. Crit Rev Clin Lab Sci. (2019) 56:458–71. doi: 10.1080/10408363.2019.1641789 31393193

[31] Sayed S Cherniak W Lawler M Tan SY El Sadr W Wolf N . Improving pathology and laboratory medicine in low-income and middle-income countries: roadmap to solutions. Lancet. (2018) 391:1939–52. doi: 10.1016/S0140-6736(18)30459-8, PMID: 29550027

[32] Thomas RE Vaska M Naugler C Turin TC. Interventions at the laboratory level to reduce laboratory test ordering by family physicians: systematic review. Clin Biochem. (2015) 48:1358–65. doi: 10.1016/j.clinbiochem.2015.09.014, PMID: 26436568

[33] Stroup DF Berlin JA Morton SC Olkin I Williamson GD Rennie D . Meta-analysis of observational studies in epidemiology: a proposal for reporting. Meta-analysis of Observational Studies in Epidemiology (MOOSE) group. JAMA. (2000) 283:2008–12. doi: 10.1001/jama.283.15.2008, PMID: 10789670

[34] Page MJ McKenzie JE Bossuyt PM Boutron I Hoffmann TC Mulrow CD . The PRISMA 2020 statement: an updated guideline for reporting systematic reviews. BMJ. (2021) 372:n71. doi: 10.1136/bmj.n71 33782057 PMC8005924

[35] Wells G Shea BJ O’Connell D Peterson J Welch V Losos M . The Newcastle–Ottawa scale (NOS) for assessing the quality of non-randomized studies in meta-analyses. Ottawa: Ottawa Hospital Research Institute (2014) Available at: http://www.ohri.ca/programs/clinical_epidemiology/oxford.asp.

[36] Higgins JPT Li T Deeks JJ. Chapter 6: choosing effect measures and computing estimates of effect In: Higgins JPT Thomas J Chandler J Cumpston M Li T Page MJ , editors. Cochrane handbook for systematic reviews of interventions: Cochrane (2020) Available at: www.training.cochrane.org/handbook

[37] Knapp G Hartung J. Improved tests for a random effects meta-regression with a single covariate. Stat Med. (2003) 22:2693–710. doi: 10.1002/sim.1482, PMID: 12939780

[38] IntHout J Ioannidis JP Borm GF. The Hartung-Knapp–Sidik-Jonkman method for random effects meta-analysis is straightforward and considerably outperforms the standard DerSimonian–Laird method. BMC Med Res Methodol. (2014) 14:25. doi: 10.1186/1471-2288-14-25, PMID: 24548571 PMC4015721

[39] Veroniki AA Jackson D Viechtbauer W Bender R Bowden J Knapp G . Methods to estimate the between-study variance and its uncertainty in meta-analysis. Res Synth Methods. (2016) 7:55–79. doi: 10.1002/jrsm.1164, PMID: 26332144 PMC4950030

[40] Higgins JP Thompson SG. Quantifying heterogeneity in a meta-analysis. Stat Med. (2002) 21:1539–58. doi: 10.1002/sim.1186, PMID: 12111919

[41] IntHout J Ioannidis JP Rovers MM Goeman JJ. Plea for routinely presenting prediction intervals in meta-analysis. BMJ Open. (2016) 6:e010247. doi: 10.1136/bmjopen-2015-010247, PMID: 27406637 PMC4947751

[42] Harrer M Cuijpers P Furukawa Toshi A Ebert DD. Doing meta-analysis with R: A hands-on guide. 1st ed. Boca Raton, FL: Chapman & Hall/CRC Press (2021).

[43] Viechtbauer W Cheung MW. Outlier and influence diagnostics for meta-analysis. Res Synth Methods. (2010) 1:112–25. doi: 10.1002/jrsm.11 26061377

[44] Egger M Davey Smith G Schneider M Minder C. Bias in meta-analysis detected by a simple, graphical test. BMJ. (1997) 315:629–34. doi: 10.1136/bmj.315.7109.629, PMID: 9310563 PMC2127453

[45] R Core Team . R: A language and environment for statistical computing. Vienna, Austria: R Foundation for Statistical Computing (2022) Available at: https://www.R-project.org/.

[46] Schwarzer G . (2022). meta: General package for meta-analysis. Available at: https://github.com/guido-s/meta/

[47] Cuijpers P Furukawa T Ebert DD. (2022). dmetar: Companion R package for the guide doing meta-analysis in R. Available at: https://dmetar.protectlab.org

[48] Bulbul M Uckardes F Karacor T Nacar MC Kaplan S Kirici P . Can complete blood count parameters that change according to trimester in pregnancy be used to predict severe preeclampsia? J Obstet Gynaecol. (2021) 41:1192–8. doi: 10.1080/01443615.2020.1854697, PMID: 33645411

[49] Oğlak SC Tunç Ş Ölmez F. First trimester mean platelet volume, neutrophil to lymphocyte ratio, and platelet to lymphocyte ratio values are useful markers for predicting preeclampsia. Ochsner J. (2021) 21:364–70. doi: 10.31486/toj.21.0026 34984051 PMC8675624

[50] Kirbas A Ersoy AO Daglar K Dikici T Biberoglu EH Kirbas O . Prediction of preeclampsia by first trimester combined test and simple complete blood count parameters. J Clin Diagn Res. (2015) 9:QC20–3. doi: 10.7860/JCDR/2015/15397.6833, PMID: 26674673 PMC4668480

[51] Gezer C Ekin A Ertas IE Ozeren M Solmaz U Mat E . High first-trimester neutrophil-to-lymphocyte and platelet-to-lymphocyte ratios are indicators for early diagnosis of preeclampsia. Ginekol Pol. (2016) 87:431–5. doi: 10.5603/GP.2016.0021, PMID: 27418220

[52] Mannaerts D Faes E Goovaerts I Stoop T Cornette J Gyselaers W . Flow-mediated dilation and peripheral arterial tonometry are disturbed in preeclampsia and reflect different aspects of endothelial function. Am J Physiol Regul Integr Comp Physiol. (2017) 313:R518–25. doi: 10.1152/ajpregu.00514.2016, PMID: 28794106

[53] Hale AL Karaca N Akpak YK Arslan E. The role of hematological and biochemical markers in preeclampsia prediction. J Clin Anal Med. (2017) 8:306–9. doi: 10.4328/JCAM.5033

[54] Mannaerts D Heyvaert S De Cordt C Macken C Loos C Jacquemyn Y. Are neutrophil/lymphocyte ratio (NLR), platelet/lymphocyte ratio (PLR), and/or mean platelet volume (MPV) clinically useful as predictive parameters for preeclampsia? J Matern Fetal Neonatal Med. (2019) 32:1412–9. doi: 10.1080/14767058.2017.1410701, PMID: 29179639

[55] Vilotić A Nacka-Aleksić M Pirković A Bojić-Trbojević Ž Dekanski D Jovanović KM. IL-6 and IL-8: an overview of their roles in healthy and pathological pregnancies. Int J Mol Sci. (2022) 23:14574. doi: 10.3390/ijms232314574, PMID: 36498901 PMC9738067

[56] Saito S . Cytokine network at the feto-maternal Interface. J Reprod Immunol. (2000) 47:87–103. doi: 10.1016/S0165-0378(00)00060-7, PMID: 10924744

[57] Walsh SW Nugent WH Archer KJ Al Dulaimi M Washington SL Strauss JF 3rd. Epigenetic regulation of interleukin-17-related genes and their potential roles in neutrophil vascular infiltration in preeclampsia. Reprod Sci. (2022) 29:154–62. doi: 10.1007/s43032-021-00605-3, PMID: 33959890 PMC8571121

[58] Aneman I Pienaar D Suvakov S Simic TP Garovic VD McClements L. Mechanisms of key innate immune cells in early- and late-onset preeclampsia. Front Immunol. (2020) 11:1864. doi: 10.3389/fimmu.2020.01864, PMID: 33013837 PMC7462000

[59] Arici A Seli E Senturk LM Gutierrez LS Oral E Taylor HS. Interleukin-8 in the human endometrium. J Clin Endocrinol Metab. (1998) 83:1783–7. doi: 10.1210/jcem.83.5.4754 9589693

[60] Corre I Pineau D Hermouet S. Interleukin-8: an autocrine/paracrine growth factor for human hematopoietic progenitors acting in synergy with colony stimulating factor-1 to promote monocyte-macrophage growth and differentiation. Exp Hematol. (1999) 27:28–36. doi: 10.1016/S0301-472X(98)00032-0 9923441

[61] Bellos I Karageorgiou V Kapnias D Karamanli K-E Siristatidis C. The role of interleukins in preeclampsia: a comprehensive review. Am J Reprod Immunol. (2018) 80:e13055. doi: 10.1111/aji.13055, PMID: 30265415

[62] Walsh SW Nugent WH Al Dulaimi M Washington SL Dacha P Strauss JF 3rd. Proteases activate pregnancy neutrophils by a protease-activated receptor 1 pathway: epigenetic implications for preeclampsia. Reprod Sci. (2020) 27:2115–27. doi: 10.1007/s43032-020-00232-4, PMID: 32542542 PMC7529957

[63] Ding W Chim SSC Wang CC Lau CSL Leung TY. Molecular mechanism and pathways of normal human parturition in different gestational tissues: a systematic review of transcriptome studies. Front Physiol. (2021) 12:730030. doi: 10.3389/fphys.2021.730030, PMID: 34566691 PMC8461075

[64] Ding W Lau SL Wang CC Zhang T Getsko O Lee NMW . Dynamic changes in maternal immune biomarkers during labor in nulliparous vs multiparous women. Am J Obstet Gynecol. (2022) 227:627.e1–627.e23. doi: 10.1016/j.ajog.2022.05.036, PMID: 35609644

[65] Willems J Joniau M Cinque S van Damme J. Human granulocyte chemotactic peptide (IL-8) as a specific neutrophil degranulator: comparison with other monokines. Immunology. (1989) 67:540–2. PMID: 2670752 PMC1385329

[66] Wang Y Gu Y Alexander JS Lewis DF. Preeclampsia status controls interleukin-6 and soluble IL-6 receptor release from neutrophils and endothelial cells: relevance to increased inflammatory responses. Pathophysiology. (2021) 28:202–11. doi: 10.3390/pathophysiology28020013, PMID: 35366257 PMC8830466

[67] Cemgil Arikan D Aral M Coskun A Ozer A. Plasma IL-4, IL-8, IL-12, interferon-γ and CRP levels in pregnant women with preeclampsia, and their relation with severity of disease and fetal birth weight. J Matern Fetal Neonatal Med. (2012) 25:1569–73. doi: 10.3109/14767058.2011.648233, PMID: 22185464

[68] Matsubara K Ochi H Kitagawa H Yamanaka K Kusanagi Y Ito M. Concentrations of serum granulocyte-colony-stimulating factor in normal pregnancy and preeclampsia. Hypertens Pregnancy. (1999) 18:95–106. doi: 10.3109/10641959909009614 10464003

[69] Hayashi M Hamada Y Ohkura T. Elevation of granulocyte-macrophage colony-stimulating factor in the placenta and blood in preeclampsia. Am J Obstet Gynecol. (2004) 190:456–61. doi: 10.1016/j.ajog.2003.07.032, PMID: 14981389

[70] Petrakis V Panagopoulos P Trypsianis G Papazoglou D Papanas N. Fasting plasma glucose increase and neutrophil to lymphocyte ratio (NLR) as risk predictors of clinical outcome of COVID-19 pneumonia in type 2 diabetes mellitus. Exp Clin Endocrinol Diabetes. (2023) 131:194–7. doi: 10.1055/a-2009-6937 36623835

[71] Wei T Li J Cheng Z Jiang L Zhang J Wang H . Hematological characteristics of COVID-19 patients with fever infected by the omicron variant in Shanghai: a retrospective cohort study in China. J Clin Lab Anal. (2023) 37:e24808. doi: 10.1002/jcla.24808, PMID: 36525342 PMC9833982

[72] Retief CA Retief HJ Van der Merwe S. Evaluating the neutrophil-to-lymphocyte ratio as an indicator for early referral of patients with COVID-19 pneumonia to a high-care facility. S Afr Med J. (2022) 112:795–9. doi: 10.7196/SAMJ.2022.v112i10.16590 36472334

[73] Fernandes NF Costa IF Pereira KN de Carvalho JAM Paniz C. Hematological ratios in coronavirus disease 2019 patients with and without invasive mechanical ventilation. J Investig Med. (2023) 71:321–8. doi: 10.1177/10815589221149189, PMID: 36680362

[74] Arslan B Bicer IG Sahin T Vay M Dilek O Destegul E. Clinical characteristics and hematological parameters associated with disease severity in COVID-19 positive pregnant women undergoing cesarean section: a single-center experience. J Obstet Gynaecol Res. (2022) 48:402–10. doi: 10.1111/jog.15108, PMID: 34837446 PMC9011896

[75] Aldika Akbar MI Gumilar KE Rahestyningtyas E Wardhana MP Mulawardhana P Anas JY . Accuracy of screening methods of COVID-19 in pregnancy: a cohort study. Minerva Obstet Gynecol. (2023) 75:117–25. doi: 10.23736/S2724-606X.21.04979-4 34851075

[76] Aydin Güzey N Uyar TE. Evaluation of 254 cesarean sections with COVID-19 in terms of anesthesia and clinical course: 1-year experience. J Anesth. (2022) 36:514–23. doi: 10.1007/s00540-022-03086-z, PMID: 35691987 PMC9188761

[77] Supák D Mészáros B Nagy M Gáspár D Wagner LJ Kukor Z . Case report: COVID-19 infection in a pregnant 33-year-old kidney transplant recipient. Front Med. (2022) 9:948025. doi: 10.3389/fmed.2022.948025, PMID: 36111115 PMC9468219

[78] Supák D Mészáros B Turi B Herold Z Kukor Z Valent S. Predicting potentially fatal COVID-19 disease in pregnant patients using the neutrophil-to-lymphocyte ratio (NLR). J Clin Med. (2023) 12:6896. doi: 10.3390/jcm12216896, PMID: 37959361 PMC10649139

[79] Lurie S Frenkel E Tuvbin Y. Comparison of the differential distribution of leukocytes in preeclampsia versus uncomplicated pregnancy. Gynecol Obstet Investig. (1998) 45:229–31. doi: 10.1159/000009973, PMID: 9623786

[80] Kurtoglu E Kokcu A Celik H Tosun M Malatyalioglu E. May ratio of neutrophil to lymphocyte be useful in predicting the risk of developing preeclampsia? A pilot study. J Matern Fetal Neonatal Med. (2015) 28:97–9. doi: 10.3109/14767058.2014.905910, PMID: 24635498

[81] Aslan MM Yeler MT Yuvacı HU Cerci IA Cevrioğlu AS Ozden S. Can the neutrophil-to-lymphocyte ratio (NLR) predicts fetal loss in preeclampsia with severe features? Pregnancy Hypertens. (2020) 22:14–6. doi: 10.1016/j.preghy.2020.07.005, PMID: 32693328

[82] Çintesun E Incesu Çintesun FN Ezveci H Akyürek F Çelik Ç. Systemic inflammatory response markers in preeclampsia. J Lab Physicians. (2018) 10:316–9. doi: 10.4103/JLP.JLP_144_17, PMID: 30078969 PMC6052816

[83] Bokslag A van Weissenbruch M Mol BW de Groot CJ. Preeclampsia; short and long-term consequences for mother and neonate. Early Hum Dev. (2016) 102:47–50. doi: 10.1016/j.earlhumdev.2016.09.007 27659865

[84] Ma’ayeh M Costantine MM. Prevention of preeclampsia. Semin Fetal Neonatal Med. (2020) 25:101123. doi: 10.1016/j.siny.2020.101123, PMID: 32513597 PMC8236336

[85] Yang Y Le Ray I Zhu J Zhang J Hua J Reilly M. Preeclampsia prevalence, risk factors, and pregnancy outcomes in Sweden and China. JAMA Netw Open. (2021) 4:e218401. doi: 10.1001/jamanetworkopen.2021.8401, PMID: 33970258 PMC8111481

[86] Bartsch E Medcalf KE Park AL Ray JG High Risk of Pre-Eclampsia Identification Group. Clinical risk factors for pre-eclampsia determined in early pregnancy: systematic review and meta-analysis of large cohort studies. BMJ. (2016) 353:i1753. doi: 10.1136/bmj.i1753, PMID: 27094586 PMC4837230

[87] Hamzah STR Aminuddin II Rachmat M. Antenatal care parameters that are the risk factors in the event of preeclampsia in primigravida. Gac Sanit. (2021) 35:S263–7. doi: 10.1016/j.gaceta.2021.10.073, PMID: 34929827

[88] Poon LC Shennan A Hyett JA Kapur A Hadar E Divakar H . The International Federation of Gynecology and Obstetrics (FIGO) initiative on pre-eclampsia: a pragmatic guide for first-trimester screening and prevention. Int J Gynaecol Obstet. (2019) 146:390–1. doi: 10.1002/ijgo.12892, PMID: 31111484 PMC6944283

[89] Kay VR Wedel N Smith GN. Family history of hypertension, cardiovascular disease, or diabetes and risk of developing preeclampsia: a systematic review. J Obstet Gynaecol Can. (2021) 43:227–236.e19. doi: 10.1016/j.jogc.2020.08.010 33268309

[90] Gestational hypertension and preeclampsia: ACOG practice bulletin summary, number 222. Obstet Gynecol. (2020) 135:1492–5. doi: 10.1097/AOG.0000000000003892, PMID: 32443077

[91] MacDonald TM Walker SP Hannan NJ Tong S Kaitu’u-Lino TJ. Clinical tools and biomarkers to predict preeclampsia. EBioMedicine. (2022) 75:103780. doi: 10.1016/j.ebiom.2021.103780, PMID: 34954654 PMC8718967

[92] Skråstad RB Hov GG Blaas HG Romundstad PR Salvesen KÅ. A prospective study of screening for hypertensive disorders of pregnancy at 11–13 weeks in a Scandinavian population. Acta Obstet Gynecol Scand. (2014) 93:1238–47. doi: 10.1111/aogs.12479, PMID: 25146367

[93] Huang T Bedford HM Rashid S Rasasakaram E Priston M Mak-Tam E . Modified multiple marker aneuploidy screening as a primary screening test for preeclampsia. BMC Pregnancy Childbirth. (2022) 22:190. doi: 10.1186/s12884-022-04514-4, PMID: 35260099 PMC8903171

[94] Rana S Lemoine E Granger JP Karumanchi SA. Preeclampsia: pathophysiology, challenges, and perspectives. Circ Res. (2019) 124:1094–112. doi: 10.1161/CIRCRESAHA.118.313276 30920918

[95] Lin TY Huang HY Chan KS Chen YT Chu FC Shaw SW. Current update of first trimester preeclampsia screening in Asia. J Obstet Gynaecol Res. (2021) 47:26–33. doi: 10.1111/jog.14524, PMID: 33063401

[96] Mosimann B Amylidi-Mohr SK Surbek D Raio L. First trimester screening for preeclampsia—a systematic review. Hypertens Pregnancy. (2020) 39:1–11. doi: 10.1080/10641955.2019.1682009 31670986

[97] Acestor N Goett J Lee A Herrick TM Engelbrecht SM Harner-Jay CM . Towards biomarker-based tests that can facilitate decisions about prevention and management of preeclampsia in low-resource settings. Clin Chem Lab Med. (2016) 54:17–27. doi: 10.1515/cclm-2015-0069 25992513

[98] Rolnik DL Nicolaides KH Poon LC. Prevention of preeclampsia with aspirin. Am J Obstet Gynecol. (2022) 226:S1108–19. doi: 10.1016/j.ajog.2020.08.045 32835720

[99] Huai J Lin L Juan J Chen J Li B Zhu Y . Preventive effect of aspirin on preeclampsia in high-risk pregnant women with stage 1 hypertension. J Clin Hypertens. (2021) 23:1060–7. doi: 10.1111/jch.14149, PMID: 33400389 PMC8678830

[100] Lin L Huai J Li B Zhu Y Juan J Zhang M . A randomized controlled trial of low-dose aspirin for the prevention of preeclampsia in women at high risk in China. Am J Obstet Gynecol. (2022) 226:251.e1–251.e12. doi: 10.1016/j.ajog.2021.08.004 34389292

[101] Cruz-Lemini M Vázquez JC Ullmo J Llurba E. Low-molecular-weight heparin for prevention of preeclampsia and other placenta-mediated complications: a systematic review and meta-analysis. Am J Obstet Gynecol. (2022) 226:S1126–S1144.e17. doi: 10.1016/j.ajog.2020.11.006, PMID: 34301348

[102] Mészáros B Veres DS Nagyistók L Somogyi A Rosta K Herold Z . Pravastatin in preeclampsia: a meta-analysis and systematic review. Front Med. (2023) 9:2023. doi: 10.3389/fmed.2022.1076372, PMID: 36714131 PMC9880057

[103] Khaing W Vallibhakara SA Tantrakul V Vallibhakara O Rattanasiri S McEvoy M . Calcium and vitamin D supplementation for prevention of preeclampsia: a systematic review and network meta-analysis. Nutrients. (2017) 9:1141. doi: 10.3390/nu9101141, PMID: 29057843 PMC5691757

[104] Poniedziałek-Czajkowska E Mierzyński R. Could vitamin D be effective in prevention of preeclampsia? Nutrients. (2021) 13:3854. doi: 10.3390/nu13113854, PMID: 34836111 PMC8621759

[105] Patrelli TS Dall’Asta A Gizzo S Pedrazzi G Piantelli G Jasonni VM . Calcium supplementation and prevention of preeclampsia: a meta-analysis. J Matern Fetal Neonatal Med. (2012) 25:2570–4. doi: 10.3109/14767058.2012.715220 22889274

[106] Fogacci S Fogacci F Banach M Michos ED Hernandez AV Lip GYH . Vitamin D supplementation and incident preeclampsia: a systematic review and meta-analysis of randomized clinical trials. Clin Nutr. (2020) 39:1742–52. doi: 10.1016/j.clnu.2019.08.015, PMID: 31526611

[107] Yuan J Yu Y Zhu T Lin X Jing X Zhang J. Oral magnesium supplementation for the prevention of preeclampsia: a meta-analysis or randomized controlled trials. Biol Trace Elem Res. (2022) 200:3572–81. doi: 10.1007/s12011-021-02976-9 34775542

[108] Ozimek JA Kilpatrick SJ. Maternal mortality in the twenty-first century. Obstet Gynecol Clin N Am. (2018) 45:175–86. doi: 10.1016/j.ogc.2018.01.004, PMID: 29747724

[109] Collier AY Molina RL. Maternal mortality in the United States: updates on trends, causes, and solutions. NeoReviews. (2019) 20:e561–74. doi: 10.1542/neo.20-10-e561, PMID: 31575778 PMC7377107

[110] Bødker B Hvidman L Weber T Møller M Andersen BR Westergaard HB . Reduction in maternal mortality in Denmark over three decades. Dan Med J. (2021) 68:A02210143. 34477097

[111] Diguisto C Saucedo M Kallianidis A Bloemenkamp K Bødker B Buoncristiano M . Maternal mortality in eight European countries with enhanced surveillance systems: descriptive population based study. BMJ. (2022) 379:e070621. doi: 10.1136/bmj-2022-070621, PMID: 36384872 PMC9667469

[112] Stevens W Shih T Incerti D Ton TGN Lee HC Peneva D . Short-term costs of preeclampsia to the United States health care system. Am J Obstet Gynecol. (2017) 217:237–248.e16. doi: 10.1016/j.ajog.2017.04.032, PMID: 28708975

[113] Dymara-Konopka W Laskowska M Oleszczuk J. Preeclampsia—current management and future approach. Curr Pharm Biotechnol. (2018) 19:786–96. doi: 10.2174/1389201019666180925120109 30255751

[114] Ghulmiyyah L Sibai B. Maternal mortality from preeclampsia/eclampsia. Semin Perinatol. (2012) 36:56–9. doi: 10.1053/j.semperi.2011.09.011 22280867

[115] von Dadelszen P Firoz T Donnay F Gordon R Justus Hofmeyr G Lalani S . Preeclampsia in low and middle income countries-health services lessons learned from the PRE-EMPT (PRE-Eclampsia-Eclampsia Monitoring, Prevention and Treatment) project. J Obstet Gynaecol Can. (2012) 34:917–26. doi: 10.1016/S1701-2163(16)35405-6, PMID: 23067947

[116] Bossman E Johansen MA Zanaboni P. mHealth interventions to reduce maternal and child mortality in sub-Saharan Africa and southern Asia: a systematic literature review. Front Glob Womens Health. (2022) 3:942146. doi: 10.3389/fgwh.2022.942146, PMID: 36090599 PMC9453039

[117] Dasari A Jacob PM Jeyapaul S Mathew AJ Abraham VJ Cherian AG. Description and outcomes of patients with eclampsia and severe pre-eclampsia in a rural hospital in north-eastern Bihar: a retrospective study. J Family Med Prim Care. (2022) 11:6096–100. doi: 10.4103/jfmpc.jfmpc_286_22, PMID: 36618200 PMC9810891

[118] Kerboua KE . NLR: a cost-effective nomogram to guide therapeutic interventions in COVID-19. Immunol Investig. (2021) 50:92–100. doi: 10.1080/08820139.2020.1773850, PMID: 32482134

[119] Tadesse Z Bekele Bayissa A Diriba T Chernet N Tsegaye S Tsega M. Neutrophil-to-lymphocyte ratio and cut-off values as predictor of severity and mortality in COVID-19 patients in millennium COVID-19 care center, Addis Ababa, Ethiopia. Int J Gen Med. (2022) 15:6739–55. doi: 10.2147/IJGM.S375565, PMID: 36039306 PMC9419908

[120] Zhang J Zeng J Zhang L Yu X Guo J Li Z. The utility of peripheral blood leucocyte ratios as biomarkers in neonatal sepsis: a systematic review and meta-analysis. Front Pediatr. (2022) 10:908362. doi: 10.3389/fped.2022.908362, PMID: 35935369 PMC9353072

[121] Can E Hamilcikan Ş Can C. The value of neutrophil to lymphocyte ratio and platelet to lymphocyte ratio for detecting early-onset neonatal Sepsis. J Pediatr Hematol Oncol. (2018) 40:e229–32. doi: 10.1097/MPH.0000000000001059, PMID: 29219889

